# Anterior thalamic nuclei lesions in rats disrupt markers of neural plasticity in distal limbic brain regions

**DOI:** 10.1016/j.neuroscience.2012.08.027

**Published:** 2012-11-08

**Authors:** J.R. Dumont, E. Amin, G.L. Poirier, M.M. Albasser, J.P. Aggleton

**Affiliations:** School of Psychology, Cardiff University, Cardiff, Wales CF10 3AT, United Kingdom

**Keywords:** ATN, anterior thalamic lesion, Audp, primary auditory cortex, BSA, bovine serum albumin, cRdg, caudal dysgranular retrosplenial cortex, CREB, c-AMP response element binding protein, cRga, caudal granular retrosplenial cortex, area a, cRgb, caudal granular retrosplenial cortex, area b, DAB, diaminobenzidine, dSub, dorsal subiculum, GAP-43, growth associated protein43, Hpc, hippocampus, iCA1, intermediate CA1, iCA3, intermediate CA3, iDG, intermediate dentate gyrus, IEG, immediate early gene, IL, infralimbic cortex, lEnto, lateral entorhinal cortex, mEnt, medial entorhinal cortex, NMDA, *N*-methyl-d-aspartic acid, PBS, phosphate buffer saline, PBST, PBS containing 0.2% Triton X-100, pCREB, phosphorylated CREB, PFA, 4% paraformaldehyde in 0.1 M PBS, PL, prelimbic cortex, Prh, perirhinal cortex, pSub, postsubiculum, rRdg, rostral dysgranular retrosplenial cortex, rRgb, rostral granular retrosplenial cortex, area b, tCA, temporal CA1, tCA3, temporal CA3, CREB, GAP-43, hippocampus, phosphorylated CREB, retrosplenial cortex, *zif268*

## Abstract

In two related experiments, neurotoxic lesions were placed in the anterior thalamic nuclei of adult rats. The rats were then trained on behavioral tasks, immediately followed by the immunohistochemical measurement of molecules linked to neural plasticity. These measurements were made in limbic sites including the retrosplenial cortex, the hippocampal formation, and parahippocampal areas. In Experiment 1, rats with unilateral anterior thalamic lesions explored either novel or familiar objects prior to analysis of the immediate-early gene *zif268*. The lesions reduced *zif268* activity in the granular retrosplenial cortex and postsubiculum. Exploring novel objects resulted in local changes of hippocampal *zif268*, but this change was not moderated by anterior thalamic lesions. In Experiment 2, rats that had received either bilateral anterior thalamic lesions or control surgeries were exposed to novel room cues while running in the arms of a radial maze. In addition to *zif268*, measurements of c-AMP response element binding protein (CREB), phosphorylated CREB (pCREB), and growth associated protein43 (GAP-43) were made. As before, anterior thalamic lesions reduced *zif268* in retrosplenial cortex and postsubiculum, but there were also reductions of pCREB in granular retrosplenial cortex. Again, the hippocampus did not show lesion-induced changes in *zif268*, but there were differential effects on CREB and pCREB consistent with reduced levels of hippocampal CREB phosphorylation following anterior thalamic damage. No changes in GAP-43 were detected. The results not only point to changes in several limbic sites (retrosplenial cortex and hippocampus) following anterior thalamic damage, but also indicate that these changes include decreased levels of pCREB. As pCREB is required for neuronal plasticity, partly because of its regulation of immediate early-gene expression, the present findings reinforce the concept of an ‘extended hippocampal system’ in which hippocampal function is dependent on distal sites such as the anterior thalamic nuclei.

## Highlights

► Anterior thalamic lesions alter the ratio of hippocampal CREB to pCREB. ► Anterior thalamic lesions reduced pCREB in granular retrosplenial cortex. ► Changes in limbic pCREB may explain learning deficits after anterior thalamic lesions. ► *zif268* Hypoactivity in granular retrosplenial cortex after anterior thalamic lesions. ► Hippocampal *zif268* levels relatively insensitive to anterior thalamic damage.

## Introduction

Behavioral experiments have shown that anterior thalamic–hippocampal interactions are critical for spatial and contextual memory (Parker and Gaffan, 1997a,b; Aggleton and Brown, 1999; Warburton et al., 2000, 2001; Henry et al., 2004). Clinical studies add further weight to the notion that the anterior thalamic nuclei comprise part of an extended-hippocampal system that supports key elements of episodic memory (Van der Werf et al., 2003; Tsivilis et al., 2008; Aggleton et al., 2011; Carlesimo et al., 2011). The present study sought to understand why the anterior thalamic nuclei and hippocampus are often interdependent. For this reason, two related experiments examined the impact of anterior thalamic lesions on the activity of the hippocampus and related limbic regions by assaying levels of various molecules linked to neuronal plasticity.

Previous studies have shown how anterior thalamic lesions can reduce activity of the immediate early gene (IEG) *c-fos* within the hippocampus (Jenkins et al., 2002a,b; see also Vann and Albasser, 2009), potentially helping to explain why such lesions disrupt spatial memory tasks (see Liu et al., 2012). In addition, medial diencephalic pathology that includes the anterior thalamic nuclei disrupts cholinergic activity in the hippocampus (Savage et al., 2003; Roland and Savage, 2007). The present study pursued this inter-relationship by comparing the consequences of lesions in the anterior thalamic nuclei on activity levels of another IEG, *zif268* (also known as *egr-1* or *krox24*). The IEG *zif268* was studied as its expression is often closely associated with hippocampal plasticity and so it appears to be involved in spatial learning and memory (Richardson et al., 1992; Abraham et al., 1993, 1994; Herdegen and Leah, 1998; Tischmeyer and Grimm, 1999; Jones et al., 2001; Guzowski, 2002; Davis et al., 2003; Lindecke et al., 2006; Kubik et al., 2007; Poirier et al., 2008a). Previous studies have shown that anterior thalamic lesions lower levels of both c-*fos* and *zif268* in the retrosplenial cortex (Jenkins et al., 2004b; see also Albasser et al., 2007), suggesting that anterior thalamic lesions will also cause *zif268* hypoactivity in the hippocampus, i.e., *zif268* will again follow the pattern seen with c-*fos*. Reflecting this focus on the hippocampus, other closely related sites, e.g., the retrosplenial and parahippocampal cortices (Diana et al., 2007; Vann et al., 2009) were also examined.

In Experiment 1, all rats received unilateral anterior thalamic lesions to allow inter-hemispheric comparisons of *zif268* activity levels. These rats were divided into two groups (Group Novel and Group Familiar). One group was given novel objects to explore on the final test session before IEG analysis while the other group received just familiar objects to explore. Based on previous measures of c-*fos* expression using this protocol (Albasser et al., 2010b) it was expected that the Group Novel rats would show higher perirhinal and hippocampal IEG expression in the intact hemisphere than Group Familiar, potentially making it easier to detect any impact of anterior thalamic damage on limbic activation.

The lack of hippocampal *zif268* changes after unilateral anterior thalamic damage in Experiment 1 prompted a second experiment. In Experiment 2, the rats received bilateral anterior thalamic lesions, so that any null result would not be due to cross-hemispheric connections. This change meant that a separate surgical control group was required. The animals were also given a different behavioral task immediately prior to IEG analysis, the task more explicitly involving spatial learning. Experiment 2 also broadened the search for hippocampal-related activity changes after anterior thalamic lesions by looking at three additional molecules, as well as *zif268*. These additional targets were: (1) c-AMP response element binding protein (CREB), (2) phosphorylated CREB (pCREB), and (3) growth associated protein43 (GAP-43). The first two molecular targets were selected as there is a wealth of evidence highlighting the importance of the conversion of CREB to pCREB for the consolidation of new learning, including hippocampal-dependent learning (Silva et al., 1998; Guzowski et al., 2001; Guzowski, 2002; Mizuno et al., 2002; Winograd and Viola, 2004; Countryman et al., 2005; Warburton et al., 2005). As part of its functions, pCREB is a transcription factor for the induction of *c-fos* and *zif268* (Herdegen and Leah, 1998; Silva et al., 1998; Davis et al., 2003). For these reasons both CREB and pCREB were examined.

The final target, GAP-43, was selected as it is a regulator of growth cone motility that has been repeatedly associated with neuronal plasticity, e.g., its expression often changes in neurons undergoing neural growth (Gionotti et al., 1992; Benowitz and Routtenberg, 1997; Carmichael et al., 2005). Furthermore, GAP-43 is upregulated following axonal injury and is regarded as a key component of the systems controlling axonal regeneration (Plunet et al., 2002; Snider et al., 2002). Previous studies have established that lesions in the anterior thalamic nuclei can have chronic distal effects on c-*fos* and *zif268* levels in the retrosplenial cortex, yet not cause overt structural changes in the same region (Jenkins et al., 2004b; Poirier et al., 2008b; Poirier and Aggleton, 2009). For this reason, GAP-43 is of particular interest as any changes in this protein might reflect chronic responses to deafferentation that are not revealed by standard histological methods. Indeed, previous studies have found that hippocampal changes in GAP-43 can be observed for at least 70 days after entorhinal cortex lesions (Steward, 1995; Hardman et al., 1997).

## Experiment 1

Levels of *zif268* expression were analyzed immunohistologically in rats with unilateral anterior thalamic lesions. The rats were divided into two groups (Novel, Familiar) according to the nature of their behavioral training. The principal difference between Group Novel and Group Familiar was whether the rats had explored novel or familiar objects during the final session prior to immunohistochemical analysis.

### Materials and methods

#### Subjects

The subjects were 25 male Lister Hooded rats (*Rattus norvegicus*) housed in a 12-h light/dark cycle and weighing 270–320 g at the beginning of the experiment (Harlan, Bicester, UK). Water was provided *ad libitum* throughout, but the rats were maintained at 85% of their free-feeding weight for the duration of the experiment. The rats were divided into two groups: Novel (*n* = 13) and Familiar (*n* = 12), and where possible housed in pairs of one Group Novel rat and one Group Familiar rat. Both Experiments 1 and 2 were performed in accordance with the UK Animals (Scientific Procedures) Act (1986) and associated guidelines, thereby complying with APA ethical standards for the treatment and care of animals.

#### Surgery – anterior thalamic lesions

Surgery was performed under pentobarbitone sodium anesthesia (60 mg/kg i.p., Sigma–Aldrich Company Ltd., Dorset, UK). Once anesthetized, the animal was placed in the head-holder of the stereotaxic apparatus (Kopf Instruments, CA, USA) with the incisor bar adjusted to +5.0 relative to the horizontal plane. Following an incision, the scalp was retracted to expose the scull. A craniotomy was made and the dura cut to expose the cortex above the target location. Unilateral lesions were made by injecting 0.12 M *N*-methyl-d-aspartic acid (NMDA; Sigma Chemicals, UK) dissolved in sterile phosphate buffer (pH 7.4) into two separate sites within one hemisphere with the use of a 1-μl Hamilton syringe (Hamilton, Switzerland) that was attached to a moveable arm mounted on the stereotaxic frame. The other hemisphere was left intact. At each site, 0.22 μl of NMDA was injected over a period of 5 min, and the syringe was left *in situ* for an additional 4 min before being retracted. The anteroposterior (−0.3), mediolateral (±0.9 and ±1.8 from the midline), and height [dorso-ventral −7.0 (medial site), −6.3 (lateral site)] lesion coordinates were taken relative to bregma. After the injections, the incision was cleaned and sutured. A topical antibiotic powder (Aureomycin, Fort Dodge, Animal Health, Southampton, UK) was applied. The rats received glucose–saline (5 ml s.c.) for fluid replacement, and were then placed in a recovery chamber until they regained consciousness (i.e., movement and righting reflex). Rats were then administered an analgesic [paracetamol mixed with sucrose in the drinking water or Metacam (0.06 ml s.c.; 5 mg/ml meloxicam; Boehringer Ingelheim Vetmedica, Germany)]. The respiratory stimulant millophyline (0.1 ml s.c., Arnolds Veterinary Products, Shropshire, UK), the antimicrobial Baytril (2.5% in their drinking water, Bayer Ltd., Animal Health Division, Ireland), and a single low dose of diazepam (0.07 ml s.c., 5 mg/ml; CP Pharmaceuticals Ltd., UK) were administered as required to facilitate post-operative recovery.

#### Apparatus and materials

The rats were tested in a bow-tie shaped maze (120 cm long, 50 cm wide, and 50 cm high) made of steel. Each end of the maze formed a triangular arena and these arenas were joined at their apices by a narrow corridor (12 cm wide). In the center of the corridor, an opaque guillotine door could be lowered or raised by the experimenter to allow passage from one end of the bow-tie maze to the other. At the far wall of each of the triangular arenas were two food wells (3.5 cm in diameter and 2 cm deep) that were separated by a short, opaque wall that protruded 15 cm from the middle of the end wall. Objects were placed above these two food wells during the experiment. The short wall ensured that when exploring one object, the other object could not be seen. It was, however, easy for the rat to step around the end of the wall.

The study used 147 pairs of objects that differed in size, shape, color, and texture, but were without any obvious odor to the experimenter. The objects were large enough to cover the circular food wells but light enough for the rats to displace. The items were divided into seven groups of 21 pairs of objects.

#### Behavioral procedures

*Pre-training:* Behavioral training commenced 4 weeks after surgery. Animals were habituated for approximately 7 days. By the end of pre-training all rats would run from one end of the maze to the other and displace objects covering the food wells in order to obtain a reward (sucrose pellet; 45 mg; Noyes Purified Rodent Diet, Lancaster, NH, USA). On day 1, pairs of rats were placed in the maze for 20 min and allowed to explore and consume sucrose pellets scattered along the floor and in the food wells. On day 2, rats were trained individually for 10 min to run back and forth for a reward located only in the food wells. From day 3, the rats were introduced to the guillotine door that restricted their movement from one compartment to the other. On day 4, four identical wood blocks were introduced and gradually covered the food wells, so that by the end of the 10 min session, the rats would push the blocks in order to obtain the food reward. From day 5, three other pairs of objects were introduced that varied in size, shape, color, and weight; with the same two objects (one pair) covering the two wells in the same side of the apparatus. These three pairs of objects were only used during pre-training, i.e., they were not used during the experiment proper.

*Testing protocols:* All rats were tested for 13 sessions, each containing 20 trials. For Group Novel each session began (Trial 0; see Table 1) with the rat being placed in one end of the maze that contained two items over the baited food wells, a novel object (object A_1_) and a wood block that was familiar from its repeated use in pre-training. The rat was allowed to explore both objects freely, but after 1 min the guillotine door was raised allowing access to the second compartment, so starting Trial 1 (Table 1). The rat typically ran immediately to the opposite side of the maze, where again it could explore two objects that each covered a single sucrose pellet. One of these objects was novel (object B_1_) while the other was familiar as it was a duplicate of object A_1_ (object A_2_). After a minute, the guillotine door was raised again and the rat ran back to the first compartment of the maze (Trial 2) where object C_1_ (novel) and a duplicate of familiar object B_1_ (object B_2_) were presented. Following a further minute of free exploration, the guillotine door was raised again (Trial 3), and the rat ran back into the second compartment to explore a copy of the now familiar object C_1_ (object C_2_) and new object D_1_ (novel). This sequence continued for a total of 20 trials (Table 1).

All test objects covered one food pellet, which motivated the rats to run back and forth across the apparatus and approach the objects, but did not affect the validity of the preferential test of recognition as this relied on differential levels of object exploration. The rats were placed in a dark, quiet room, for 30 min prior to testing, and for 60 min at the completion of a session. After 60 min, the rats were returned to their home cages located in the colony room. All rats initially received 12 sessions, given over 6 days (one session in the morning and one in the afternoon). For Sessions 1–12, Group Novel was offered a pool of 126 items divided into six sets of 21 object duplicates. As new sets were used for each of the first six sessions it was necessary to re-use that same pool of 126 objects for the second six sessions (Sessions 7–12), though the order and pairings of individual objects changed. Animals were video recorded throughout training.

The rats in Group Familiar were trained in exactly the same way as Group Novel, except for one key difference. Group Familiar explored the same set of 21 objects on every session, i.e., on Sessions 1–12, although the order and individual pairing of objects changed across sessions. Consequently the rats in this second group should become very familiar with the individual objects.

On the final test (Session 13) both groups of rats were allowed to explore the same set of objects, with the order being matched across the two groups. The objects used were those that had been presented repeatedly to the Group Familiar rats (Sessions 1–12), i.e., all were highly familiar. In contrast, Group Novel had never encountered these objects before and so all were novel (see Table 1).

#### Analysis of behavior

Exploration of an object was defined as directing the nose at a distance of <1 cm to the object and/or touching it with the nose or the paws (including pushing). Sitting or turning around the object was not included. Likewise, behaviors such as freezing near the object (at a distance of <1 cm), chewing the object, or carrying the object in the mouth were not scored as exploration. Sessions 1, 7, and 13 (final) were scored for their exploration times.

#### Histology

All rats were perfused 90 min after completion of the final session, the time delay corresponding to the peak of *zif268* protein production after an initiating event (Zangenehpour and Chaudhuri, 2002). At the end of this delay (which the animals spent in a dark, quiet room), the animals were injected with an overdose of sodium pentobarbital (1 ml i.p., 200 mg/ml, Euthatal, Marial Animal Health Ltd., Harlow, Essex, UK) and perfused intracardially with 0.1 M phosphate buffer saline (PBS) followed by 4% paraformaldehyde in 0.1 M PBS (PFA). The brains were extracted from the skull and placed on a stirrer to postfix in PFA for 4 h, after which the brains were placed in 25% sucrose overnight. The brains were frozen on a microtome (Leica, UK) and sectioned at 40 μm in the coronal plane. One-in-five sections were mounted and stained with Cresyl Violet, a Nissl stain. The remaining sections were divided into four (one-in-five sections) series and frozen in cryoprotectant for later immunohistochemistry.

The size of the lesions in the anterior thalamic nuclei of all 25 rats was estimated from the Nissl stained tissue. The extent of each lesion was first drawn onto five equally-spaced, standard sections at different anterior–posterior levels from the atlas Paxinos and Watson (2005). These drawings were then scanned, and the area of damage was quantified using the program Analysis^D (Olympus, UK). Next, the percent damage to the entire anterior thalamus was quantified from these same standard sections.

#### *Zif268* immunohistochemistry and zif-positive cell counts

Sections were removed from the freezer, and washed for 10 min in PBS, four times. The sections were placed in 10 mM citrate buffer (pH 6) dissolved in deionized water and incubated in a water bath at 70 °C for 30 min. The sections were then washed in 0.3% hydrogen peroxide in PBS containing 0.2% Triton X-100 (PBST) for 10 min in order to block endogenous peroxidase activity, and rinsed for 10 min in PBST, four times. Afterwards, the sections were incubated at 4 °C for 48 h in PBST with rabbit polyclonal antibody for *zif268* (1:3000, C-19, Santa Cruz, Insight Biotechnologies, UK). The sections were then rinsed for 10 min in PBST, four times. Following the four washes, the sections were incubated in biotinylated goat anti-rabbit secondary antibody (diluted 1:200 in PBST; Vector Laboratories, Burlingame, CA, USA) and 1.5% normal goat serum for 2 h. The sections were washed again, and incubated for 1 h in avidin-biotinylated horseradish peroxidase complex in PBST (Elite Kit, Vector Laboratories). Next, sections were rinsed in 0.05 M Tris buffer (pH 7.4). The reaction was visualized using diaminobenzidine (DAB Substrate Kit, Vector Laboratories), and stopped by washing in cold PBS. Finally, the sections were mounted on gelatine-coated slides, dehydrated through a graded series of alcohols, and coverslipped.

Sections were viewed on a Leica DMRB microscope, and photographed using an Olympus DP70 camera. Automated counts of the stained cells were obtained using the program Analysis^D (Olympus, UK). Cell counts were taken without knowledge of group assignments and, where possible, without knowledge of lesion hemisphere. Images were gray-scaled, and the cell detection threshold was set manually. With few exceptions (e.g., a particularly lightly stained section), the threshold was the same for all sections from the same processing batch, i.e., held constant between hemispheres and between immunohistochemistry pairs. Counts of labeled nuclei in each region of interest were determined by counting those immunopositive cells that were above the detection threshold and between 5–20 μm in size. Counts were made in a frame area of 0.84 × 0.63 mm using 5× magnification. This frame size enabled all laminae to be included in one image. For larger regions (e.g., hippocampus), montages of the whole structure of interest were created in the coronal plane from multiple images. For all brain regions analyzed, between two and four sections per hemisphere were captured, depending on the size of the region. These counts were combined to give a mean result.

#### Regions of interest

**Fig. 1:**
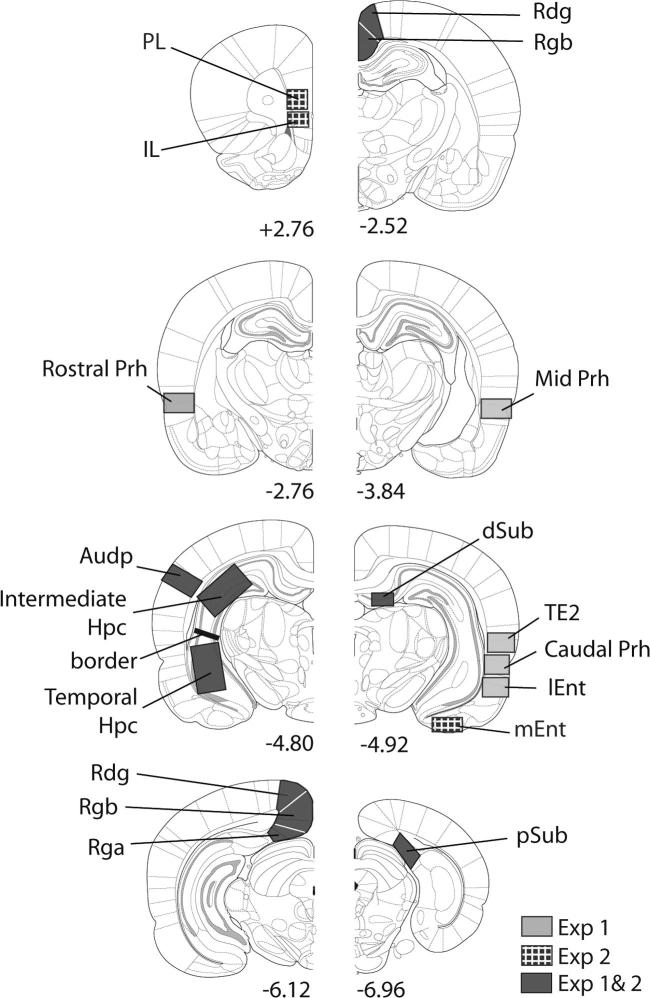
Series of coronal sections from Paxinos and Watson (2005) showing the extent of the various areas analyzed in Experiments 1 and 2. The numbers refer to anterior–posterior position relative to bregma. *Abbreviations used in the figures:* Audp, primary auditory cortex; dSub, dorsal subiculum; Hpc, hippocampus; IL, infralimbic cortex; lEnto, lateral entorhinal cortex; mEnt, medial entorhinal cortex; PL, prelimbic cortex; pSub, postsubiculum; Prh, perirhinal cortex; Rdg, dysgranular retrosplenial cortex; Rga, granular retrosplenial cortex, area a; Rgb, granular retrosplenial cortex, area b.

The various regions of interest are depicted in Fig. 1. The position and approximate coordinates of these sites are taken from images by Paxinos and Watson (2005). The hippocampus was divided into its intermediate (dorsal) and temporal (ventral) parts. Separate counts were made in CA1, CA3, and the dentate gyrus of the intermediate (dorsal) hippocampus from around the AP levels −4.80 to −5.64 from bregma (Paxinos and Watson, 2005). Counts were made in temporal (ventral) CA1 and CA3 at a similar level. The dividing border between the intermediate and temporal hippocampus corresponded to −5.0 below bregma (Paxinos and Watson, 2005). The most septal portion of the dorsal hippocampus (anterior to AP −4.80) was, however, avoided as some animals had very restricted cell loss in the ventral blade of the dentate gyrus at this level. In addition to these five hippocampal sub-regions, the dorsal subiculum and postsubiculum were counted as both are directly connected to different parts of the anterior thalamic nuclei (Meibach and Siegel, 1977; Wright et al., 2010). The lateral entorhinal cortex (from AP −4.80 to −6.30) was also included as it provides a key link between the perirhinal cortex and hippocampus (Naber et al., 1999).

Within the parahippocampal region, the perirhinal cortex was examined. The perirhinal cortex was subdivided into three rostro-caudal subregions (see Albasser et al., 2010b): rostral (from AP −2.76 to −3.84 relative to bregma), mid (AP −3.84 to −4.80), and caudal (from AP −4.80 to −6.30). Multiple counts were taken from each of these three subregions, and their means summed to provide separate totals for areas 35 and 36 (Burwell, 2001). Counts were also taken from the adjacent area TE2 (from AP −4.80 to −6.30), which has also been repeatedly implicated in novelty detection (Wan et al., 1999; Albasser et al., 2010b; Ho et al., 2011).

The retrosplenial cortex was first subdivided into granular b (Rgb), granular a (Rga), and dysgranular cortex (Rdg) (van Groen and Wyss, 1990, 1992, 2003). Separate counts were made for all three sub-regions, with areas Rdg and Rgb being further subdivided into their rostral (from AP −2.52 to −3.84) and caudal divisions (from AP −4.92 to −6.24). (This extra subdivision was not made for Rga as it is very restricted anterior to the splenium.) The retrosplenial cortex was also divided into superficial (layer II and upper layer III) and deep (lower layers III–VI) as anterior thalamic lesions can differentially affect these laminae (Jenkins et al., 2002b, 2004b; Poirier et al., 2008a,b; Poirier and Aggleton, 2009). Finally, the primary auditory cortex was examined to provide a ‘control’ region, i.e., an area where a priori there was not expected to be an effect of training condition (Novel versus Familiar) or lesion on *zif268* activity.

#### Statistics

For the initial analyses, the mean *zif268*-positive cell counts per area were calculated for each animal and separated according to hemisphere. It was then possible to compare these counts in an analysis of variance with one between factor (Group Familiar or Group Novel) and two within factors [surgery (thalamic lesion or intact hemisphere); brain site (region of interest)]. The various regions of interest were grouped, e.g., hippocampal formation, parahippocampal cortex, and retrosplenial cortex, so that each analysis of variance (ANOVA) consisted of related areas. The Greenhouse–Geisser correction was applied when the assumption of sphericity of data was violated. The analysis resulted in multiple comparisons at the regional level, and so the significance level was further adjusted using the modified Bonferroni test (Keppel, 1991) to control further for Type I errors. Therefore, for the hippocampus and retrosplenial cortex (both five subregions) the significance level was adjusted to 0.04.

Rats were paired (one Group Novel, one Group Familiar) so that the rats in each pair were trained one after the other, caged together, and perfused one after the other. This pairing made it possible to normalize data across the two behavioral groups. This normalization consisted of dividing the mean number of activated neurons in a given site of the Group Familiar animal by the combined means of the two animals in each immunohistochemistry pair (Novel and Familiar). The result of this division was then expressed as a percentage. A score of 50, therefore, corresponded to no change associated with the recognition task. Because all normalized scores across pairs of rats sum to 100 it was only necessary to consider the scores from one group (e.g., Group Familiar). Separate normalized scores were calculated for the thalamic lesion hemisphere of each Familiar:Novel pair and the control hemisphere of each Familiar:Novel pair. The effects of the lesions on task condition (novelty or familiarity) could then be examined using the normalized scores derived from the intact hemisphere compared with those from the lesioned hemisphere. As noted above, because of normalization it was only necessary to analyze the scores from one group, e.g., from Group Familiar, using a two-way within subjects ANOVA (within factors hemisphere and region). For direct site to site comparisons the normalized scores cannot be used as the total score for every site would always be 100.

### Results

#### Unilateral anterior thalamic lesions

**Fig. 2:**
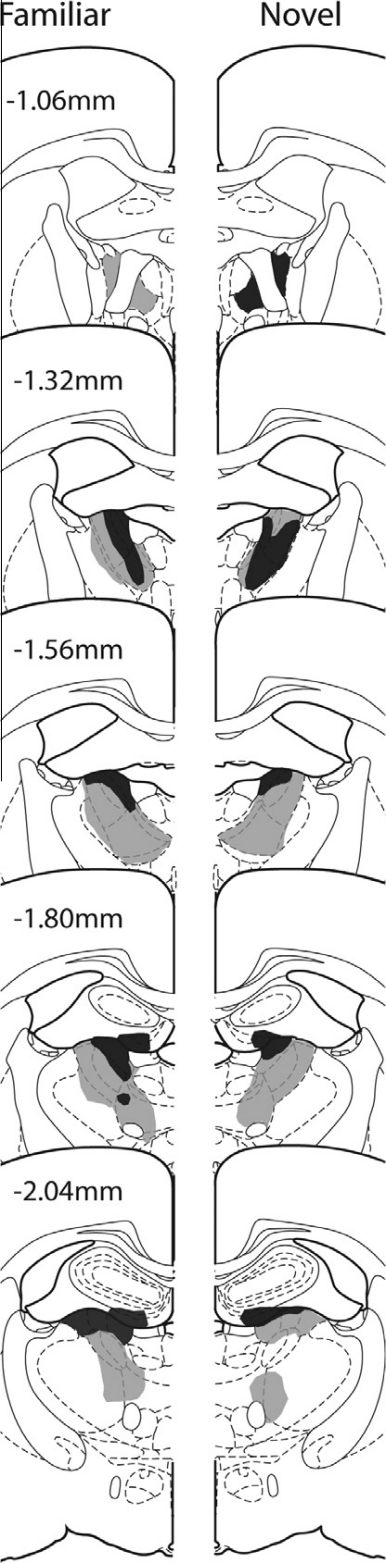
Experiment 1. Coronal sections depicting the cases with the smallest (dark gray) and largest (light gray) unilateral anterior thalamic nuclei lesions in Group Familiar (left) and Group Novel (right). The numbers refer to the approximate distance of the section in mm caudal to bregma. The sections are modified from Paxinos and Watson (2005).

Seven animals were excluded from all analyses due to the small size of their lesions (five from Group Novel and two from Group Familiar). Consequently, Group Novel comprised eight rats while Group Familiar comprised 10 rats. In these remaining 18 cases, the cell loss was centered on the anterior thalamic nuclei, which was the sole common lesion site across all cases. Fig. 2 depicts the lesions in those cases with the smallest and largest lesions within the two groups. The respective mean tissue loss across the anterior thalamic nuclei in these rats was 66.7% (Group Familiar) and 67.1% (Group Novel). The corresponding median scores were 65.6% and 69.3%, respectively. In most rats there was some sparing in the most caudal parts of these thalamic nuclei as well as the most medial portion of the anterior medial nucleus. Importantly, although the NMDA injections created discrete regions of cell loss, these regions did not extend across the midline into the opposite hemisphere. In some cases (*n* = 9), there was damage to the rostral portion of the lateral dorsal nucleus. There was also restricted cell loss in the medial blade of the septal dentate gyrus immediately dorsal to the anterior ventral nucleus in twelve of the cases. In one case there was some additional damage to the fornix. Involvement of the parataenial and reticular nuclei was observed in only the largest lesions.

#### Object recognition performance – D1 and D2 discrimination indices

Two indices of object recognition were calculated. Index D1 is the difference in time spent exploring the novel object and the familiar object. Index D2 then divides this difference (D1) by the total amount of time spent exploring both objects. Consequently, the D2 index ranges between +1 and −1. The D2 measure can better compensate for differences in overall amounts of exploration between animals. The D1 index was calculated by adding the difference data (D1) from all 20 trials (cumulative D1) while D2 then used the total exploration data from all trials (cumulative D1/total exploration). These indices were calculated for Sessions 1, 7, and 13. For Group Novel, indices D1 and D2 always reflected novelty discrimination, whereas for Group Familiar these same indices reflected relative recency discriminations for Sessions 7 and 13.

**Fig. 3:**
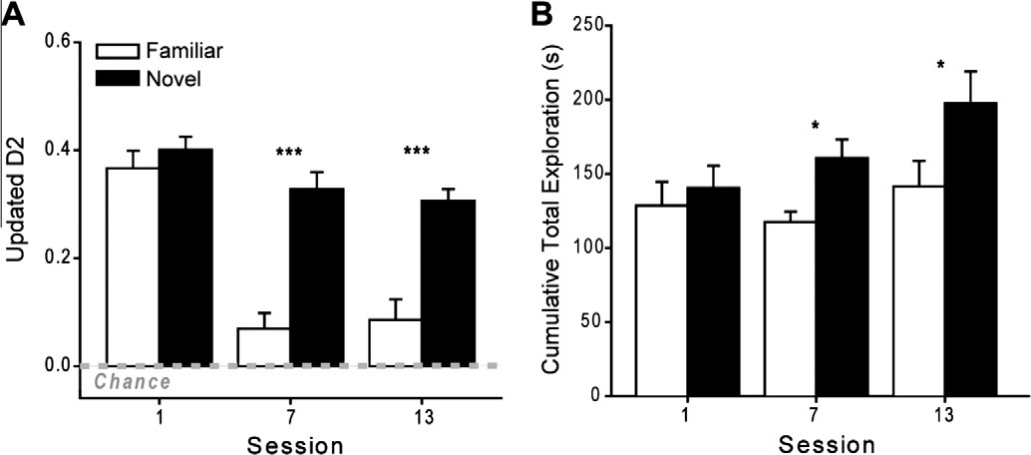
Object recognition behavior: (A) The final updated D2 scores (recognition index) are given for Group Novel and Group Familiar for Sessions 1, 7, and 13. (B) The cumulative amount of object exploration in each of Sessions 1, 7, and 13. Data shown are mean ± standard error of the mean (SEM). Group differences: ^∗^*p* < 0.05; ^∗∗∗^*p* < 0.001.

Fig. 3A shows how Group Novel continued to show a marked preference for novel objects (including Session 13) while the preference shown by Group Familiar diminished over testing as the individual objects were increasingly re-presented. Reflecting this pattern, the ANOVA with the between subject factor Group (Novel or Familiar) and the within factor Session (1, 7, and 13) revealed significant main effects of Group (D1: *F*_(1, 16)_ = 52.2, *p* < 0.001; D2: *F*_(1, 16)_ = 39.0, *p* < 0.001) and Session (D1: *F*_(2, 32)_ = 10.12, *p* < 0.001; D2: *F*_(2, 32)_ = 25.8, *p* < 0.001), as well as a Group × Session interaction (D1: *F*_(2, 32)_ = 8.10, *p* = 0.001; D2: *F*_(2, 32)_ = 8.06, *p* = 0.001) for both the D1 and D2 scores. Examination of the simple effects indicated that Group Novel and Group Familiar had comparable D1 and D2 scores for Session 1 (both *p* > 0.1). By Session 13 (final session), Group Novel spent significantly more time than Group Familiar exploring the novel items compared with the familiar ones (Fig. 3A), reflecting how the objects had become familiar for Group Familiar (D1: *F*_(1, 48)_ = 42.4, *p* < 0.001; D2: *F*_(1, 48)_ = 25.0, *p* < 0.001).

One-sample *t*-tests confirmed that both groups explored the novel objects significantly more than the familiar objects during Session 1 (D1 for Group Novel: *t*_7_ = 8.06, *p* < 0.001; D1 for Group Familiar: *t*_9_ = 7.66, *p* < 0.001; D2 for Group Novel *t*_7_ = 16.6, *p* < 0.001; D2 for Group Familiar: *t*_9_ = 11.4, *p* < 0.001), i.e., the rats recognized the novel objects. By Session 13 (final) Group Novel still spent considerably more time exploring the novel items than the familiar ones (D1: *t*_7_ = 8.33, *p* < 0.001; D2: *t*_7_ = 14.1, *p* < 0.001). Group Familiar also still showed a recency discrimination (one-sample *t* test, D1: *t*_9_ = 3.18, *p* = 0.011; D2: *t*_9_ = 2.25, *p* = 0.051), although the level of discrimination was appreciably lower than that in Group Novel.

#### Cumulative total exploration

Although the two groups initially showed comparable total levels of object exploration (Session 1, Fig. 3B), a difference emerged by the final session, reflecting the increased exploration of the novel objects (Fig. 3B). An ANOVA examining these cumulative exploration levels yielded a significant main effect of Group (*F*_(1, 16)_ = 5.38, *p* = 0.034) and Session (*F*_(2, 32)_ = 4.73, *p* = 0.016). Although the Group × Session interaction was not significant (*p* > 0.1), the simple effects revealed that on the final session Group Familiar explored the objects significantly less compared with Group Novel (*F*_(1, 48)_ = 5.98, *p* = 0.018).

#### Immediate-early gene (*zif268*) results

The *zif268* analyses involved eight Group Novel and ten Group Familiar rats. All 18 rats were included in those comparisons based on raw cell counts (between subjects). For the normalized counts (within subjects) the principal data came from pairs that had been grouped throughout training and were subsequently reacted together. This behavioral pairing was not possible in every case as those lesions that were unacceptable could only be defined post histology. Consequently, one rat from Group Familiar was added to a Familiar:Novel pairing, and all three rats immunohistochemically reacted together (i.e., as a triplicate). There were two such triplicate groups (two Group Familiar, one Group Novel), alongside the six standard pairings (one Group Familiar, one Group Novel). The data from the triplicate groups were transformed to match the overall totals for the standard pairings (i.e., for the triplicates when normalization took place, each of the two rats from Group Familiar were normalized to the same rat from Group Novel, but as the rat from Group Novel would then have two normalized scores, the mean of these two scores was used).

##### Hippocampal subfields

The initial analyses compared the raw scores of *zif268*-positive cells in five hippocampal sub-regions and compared the counts from the intact and lesioned hemispheres. Despite marked differences in the absolute *zif268* counts from region to region (*F*_(4, 60)_ = 114.1, *p* < 0.001), there were no systematic differences between the lesioned and intact hemispheres (*p* > 0.1). The overall analysis of variance (one between, two within factors) also found no evidence of a task effect (Novel versus Familiar, *p* > 0.1), and no three-way interaction between region, lesion, and task condition (*p* > 0.1).

**Fig. 4:**
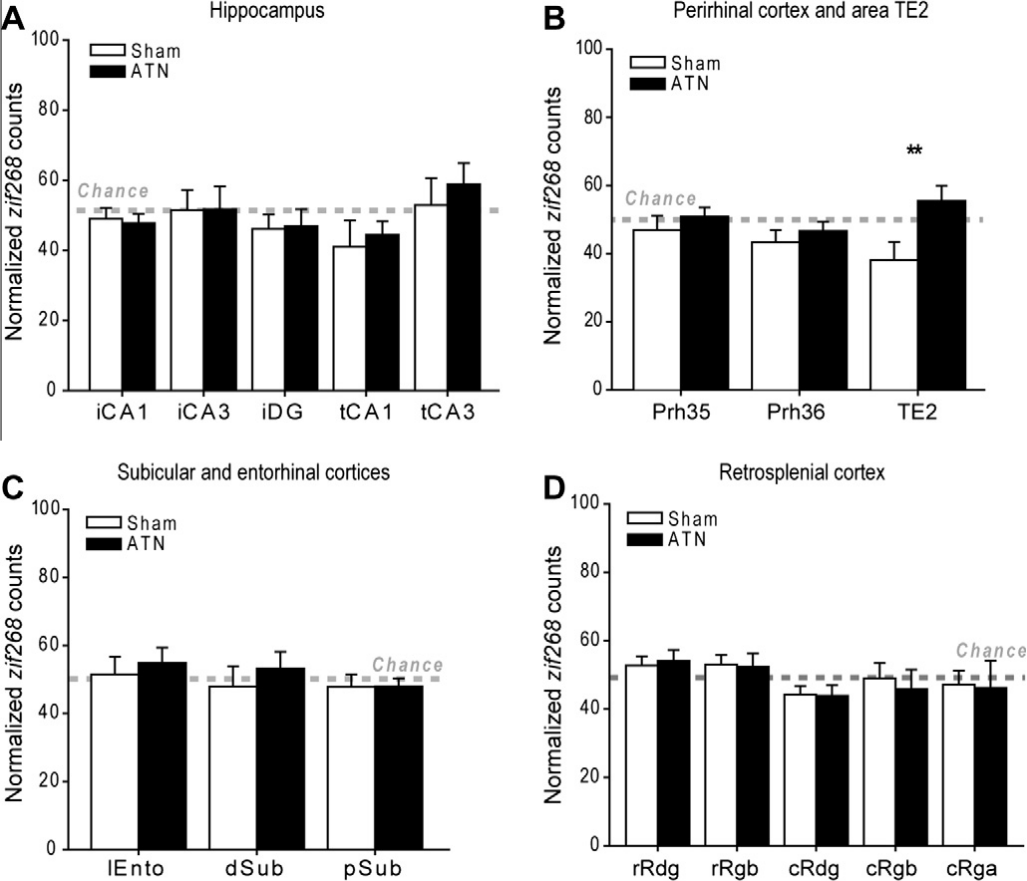
The counts of *zif268*-positive cells in different sites normalized by comparing the matched pairs of rats in Group Novel with Group Familiar, such that a score of 50 corresponds to no overall difference between the two behavioral groups (Novel and Familiar). The filled histograms display the data from the hemispheres with anterior thalamic lesions (ATN). Data shown are mean ± standard error of the mean (SEM). The results are grouped by region: (A) hippocampus, (B) perirhinal cortex and area TE2, (C) subicular and entorhinal cortices, and (D) retrosplenial cortex. *Abbreviations used in the figures:* cRdg, caudal dysgranular retrosplenial cortex; cRga, caudal granular retrosplenial cortex, area a; cRgb, caudal granular retrosplenial cortex, area b; dSub, dorsal subiculum; iCA1, intermediate CA1; iCA3, intermediate CA3; iDG, intermediate dentate gyrus; lEnto, lateral entorhinal cortex; Prh, perirhinal cortex; pSub, postsubiculum; rRdg, rostral dysgranular retrosplenial cortex; rRgb, rostral granular retrosplenial cortex, area b; tCA, temporal CA1; tCA3, temporal CA3; ^∗∗^*p* < 0.01.

The next analyses used the data normalized according to their Familiar:Novel pairings (Fig. 4A). Differential behavioral effects (Novel versus Familiar) will cause the scores to deviate from chance such that a score significantly below 50 indicates that Group Familiar had lower *zif268* counts than Group Novel. An ANOVA showed that these normalized scores differed across the regions of interest (*F*_(4, 32)_ = 3.23, *p* = 0.025), i.e., that some sub-regions had significantly different reactions to the Familiar:Novel manipulation. In particular, temporal CA1 and temporal CA3 showed differential responses, with relatively increased *zif268* scores with novel objects in CA1, but the opposite pattern in CA3. There was, however, no task by lesion interaction (*p* > 0.1), i.e., this differential response to Novel versus Familiar objects was not modulated by the anterior thalamic lesion. One-sample *t*-tests showed that none of regions (CA1, CA3) in either the sham or lesion hemisphere differed significantly from the chance score of 50 (for all analyses, *p* > 0.1).

##### Perirhinal cortex and area TE2

Although there were large differences in raw *zif268* counts (*F*_(2, 30)_ = 68.5, *p* < 0.001) across the three sub-regions (TE2 and perirhinal areas 35 and 36), there was no overall effect of lesion (*p* > 0.1) or behavioral condition (Novel versus Familiar, *p* > 0.1), and no significant interactions between these manipulations (all, *p* > 0.1). Next, the normalized scores were examined to look at the impact of the test condition and lesion status (as above, scores lower than 50 represent a lowering of the *zif268* counts in Group Familiar compared with Group Novel; Fig. 4B). In the Sham hemisphere there appeared to be a relative reduction of *zif268* in area TE2 and, to a lesser degree, in parts of the perirhinal region associated with exploring familiar objects. One-sample *t*-tests revealed that only the area TE2 scores appeared to be below chance in the control hemisphere, but this effect was not quite significant (*t*_8_ = 2.22, *p* = 0.057). These parahippocampal changes yielded a borderline overall effect of lesion (*F*_(1, 8)_ = 4.69, *p* = 0.062) but no clear site by lesion interaction (*F*_(2, 16)_ = 3.51, *p* = 0.091; Greenhouse–Geisser correction). However, an examination of the simple effects indicated that in area TE2 there was a greater reduction of *zif268* activity in Group Familiar in the intact hemisphere compared with the lesioned hemisphere (*F*_(1, 24)_ = 11.5, *p* = 0.002). It was possible to divide each of the two main perirhinal regions (areas 35 and 36) into a rostral, mid, and caudal sub-division, but this fine grained analysis did not reveal any significant changes (all *p* > 0.1).

##### Subicular and entorhinal cortices

Comparisons across the three target regions (dorsal subiculum, postsubiculum, and lateral entorhinal cortex) found raw *zif268* count differences (*F*_(2, 30)_ = 53.0, *p* < 0.001), along with evidence that the thalamic lesions reduced some IEG counts (*F*_(1, 15)_ = 4.01, *p* = 0.064). Simple effects indicated that the thalamic lesions reduced *zif268* counts in the postsubiculum (*F*_(1, 45)_ = 7.16, *p* = 0.010), although there was no site by lesion interaction (*p* > 0.1). Finally, analysis of the normalized scores failed to show that the behavioral task affected *zif268* levels in specific sites (*p* > 0.1) or that this measure was differentially affected by the surgery (*p* > 0.1; Fig. 4C).

##### Retrosplenial cortex

Consistent with previous studies, anterior thalamic lesions reduced *zif268* activity across much of the retrosplenial cortex. Consequently, analyses of the raw scores revealed an effect of lesion (*F*_(1, 16)_ = 7.94, *p* = 0.012) as well as of site (*F*_(4, 64)_ = 56.9, *p* < 0.001). There was, however, no overall effect of behavioral condition (*p* > 0.1) or any significant interactions between the above (for all *p* > 0.1). Simple effects indicated that thalamic lesions significantly reduced *zif268* counts in both rostral Rgb (*F*_(1, 80)_ = 6.38, *p* = 0.014) and caudal Rgb (*F*_(1, 80)_ = 6.99, *p* = 0.010), but there were no significant differences elsewhere (*p* > 0.1 for all). These lesion effects in Rgb were further explored by comparing the counts in the superficial and deep layers. The results indicated that for both rostral and caudal Rgb, the reduction of *zif268* activity caused by the lesion was in both the superficial layers (rostral: *F*_(1, 64)_ = 6.94, *p* = 0.011; caudal: *F*_(1, 64)_ = 6.05, *p* = 0.017) and the deep layers (rostral: *F*_(1, 64)_ = 11.2, *p* = 0.001; caudal: *F*_(1, 64)_ = 14.4, *p* < 0.001).

To examine any differential effects of the behavioral conditions, the normalized data were considered (Fig. 4D). First, there was no clear evidence that the behavioral task (Novel versus Familiar) differentially affected the various retrosplenial sub-regions (overall region effect, *p* > 0.1), and there was no interaction with lesion condition (*p* > 0.1). Inspection of the data, however, indicated that caudal dysgranular retrosplenial cortex (cRdg) had higher *zif268* counts for the novel condition in the sham hemisphere (*t*_9_ = 2.30, *p* = 0.047) with a similar, but nonsignificant, increase in *zif268* counts in the lesion hemisphere (*t*_9_ = 2.02, *p* = 0.074).

##### Control cortex (primary auditory cortex)

As expected, inspection of the raw counts in the auditory cortex did not yield a significant effect of recognition group, lesion, or a group × lesion interaction (*p* > 0.1 for all). Similarly, there was no indication that the anterior thalamic lesions modulated task performance (*p* > 0.1), or that the recognition condition influenced normalized *zif268* counts (i.e., the counts did not differ from chance for either the intact or lesion hemisphere; both *p* > 0.1).

## Experiment 2

The impact of bilateral anterior thalamic lesions on the activity of *zif268*, CREB, pCREB, and GAP-43 was examined in the same core limbic structures as Experiment 1. In addition, counts were made in two frontal brain areas, the prelimbic cortex (PL; around AP +2.76) and infralimbic cortex (IL; around AP +2.76). The choice of a spatial task prior to perfusion led to the selection of a slightly different set of parahippocampal regions, targeting regions such as the medial entorhinal cortex (mEnt) as it had previously been shown to increase IEG activity following spatial learning in this same apparatus (Wan et al., 1999; Vann et al., 2000; Aggleton et al., 2012). The control rats and those with anterior thalamic lesions were trained on a series of behavioral tasks prior to the present study (Aggleton et al., 2009). These behavioral studies showed that the anterior thalamic lesions were sufficient to impair T-maze alternation and to disrupt learning the geometric properties of a rectangular watermaze (discriminating long from short walls to identify a location). These deficits thus confirmed the effectiveness of the lesions. The same rats did, however, successfully learn a series of complex, configural visual discriminations (‘structural learning’ tasks) that were also trained in a watermaze (Aggleton et al., 2009).

Prior to perfusion, the animals received a behavioral task with the purpose of altering levels of pCREB and *zif268*. For this purpose, rats were exposed to a novel room while running up and down the arms of a radial-maze. Anterior thalamic lesions (Aggleton et al., 1996; Byatt and Dalrymple-Alford, 1996) impair working memory tasks in the radial-maze, leading to abnormal patterns of arm choice. For this reason, the experimenter rather than the rat selected the choice of arms, so matching behavioral performance across the two groups. This radial maze task took place approximately 6 months after surgery.

### Materials and methods

#### Subjects

Thirteen male rats (*R. norvegicus*) of the pigmented DA (Dark Agouti) strain (Harlan, Bicester, UK) were used in Experiment 2. All rats were housed in pairs (the additional fourteenth animal was not part of the study) under diurnal conditions, water was provided *ad libitum* throughout. The rats were maintained at 85% of their free-feeding weight for the duration of the experiment. At the time of surgery the animals were aged 4 months and weighed 220–250 g.

#### Surgery – anterior thalamic lesions

Seven naïve rats received anterior thalamic lesions (ATN2, *n* = 7). The procedure was as Experiment 1, with minor changes reflecting the smaller size of the animals. The thalamic lesions were made by injecting 0.20 μl of 0.12 NMDA (Sigma Chemicals, Poole, UK) dissolved in PBS at pH 7.2 into two sites per hemisphere using a 1-μl syringe (Hamilton, Switzerland). The stereotaxic coordinates were as follows: anterior–posterior, −0.5 from bregma; medio-lateral, 1.0 and 1.7 from the midline; dorso-ventral, −6.3 and −5.7 from the top of the dura for the medial and lateral injections, respectively. For the six surgical control rats (Sham2) a surgical needle was lowered twice per hemisphere through the cortex above the anterior thalamic nuclei, but not into subcortical regions.

#### Apparatus and materials

Testing occurred in an eight-arm radial-maze consisting of an octagonal central platform (34-cm diameter) and eight equally spaced radial arms (87 cm long, 10 cm wide). The floors of the central platform and the arms were made of wood, while panels of clear Perspex (24-cm high) formed the walls of the arms. At the end of each arm was a food well (2 cm in diameter and 0.5 cm deep). At the base of each arm was a clear Perspex guillotine door (12 cm high) that controlled access in and out of the central octagonal area. The maze was on a stand 63 cm high that could be revolved. Each door was attached to a pulley system enabling the experimenter to open and close access to each arm. The maze was placed in a rectangular room (255 cm × 330 cm × 260 cm), lit by two banks of fluorescent strip lights (0.5 m long, luminance 1022 lux) positioned over the center of the maze.

#### Behavioral procedures

Rats were trained to run down pre-selected arms of an eight-arm radial-maze to retrieve sucrose reward pellets (45 mg; Noyes Purified Rodent Diet, Lancaster, NH, USA) from the end of the arm. At the beginning of each block of eight trials, all arms were baited with a single sucrose pellet. The experimenter controlled access to each arm by using a pulley system to open the guillotine door at the start of the arm (a trial was completed once all eight arms had been visited). After all arms had been visited, the rat was contained in the central compartment of the maze for approximately 2 min while all arms were re-baited. Each animal ran one session per day over a 4 day period. Each session lasted 20 min, and consisted of multiple trials in the radial-arm maze, one after the other. Different randomized arm sequences were used throughout.

On the final test day (Session 4) the animals performed the same task as above but in an identical radial maze in a novel room. Although all animals were forced to visit the same arms in the same order, because of variations in the time it took individual rats to complete a trial, a 20-min time limit was set for each session. Each animal was placed in a holding-box in a dark, quiet room for 30 min before and 90 min after each radial-arm maze session. The holding box measured 10 cm × 10 cm × 26 cm and was made of aluminum throughout so that the lid, floor, and all walls were opaque.

#### Histology

The procedures matched those for Experiment 1, unless otherwise stated. Ninety minutes after completing the final radial-arm maze session, rats were deeply anesthetized with pentobarbitone sodium (140 mg/kg) and transcardially perfused with 0.1 M phosphate-buffer (PB) followed by 4% paraformaldehyde in 0.1 M PB containing 50 mM of sodium fluoride. The brains were rapidly removed and postfixed for 4 h in 4% paraformaldehyde, before being transferred to 30% sucrose overnight. Coronal sections were then cut at 40 μm on a freezing microtome and collected in separate dishes to undergo staining with various antibodies. Two series were collected in 0.1 M PBST for staining with *zif268* and GAP-43, and two series (one in five) were collected in 0.1 M PB containing 5% sucrose and 50 mM sodium fluoride for staining with anti-CREB and anti-pCREB. The containers with the sections for CREB and pCREB were covered in foil. A separate one-in-five series of sections was mounted directly onto gelatine-coated slides and stained using Cresyl Violet, a Nissl stain, for histological identification of specific brain regions.

#### Immunohistochemistry and cell counts

*zif268 and GAP-43:* The procedures were essentially identical to those described for *zif268* in Experiment 1 although the tissue was not placed in 10 mM citrate buffer for 30 min as this buffer can degrade the sections. The only other difference was that for GAP-43, sections were incubated in PBST containing GAP-43 rabbit polyclonal antibody [1:3000; (H-100) Santa Cruz Biotechnology, USA].

*CREB and pCREB:* Sections were incubated for 1 h in 0.1 M phosphate buffer pH 7.4 (PB) blocking serum containing 50 mM NaF, 0.3% Triton X-100 and 3% bovine serum albumin (BSA). The sections were covered at all times. Sections were next incubated in anti-CREB and anti-pCREB rabbit polyclonal antibodies (1:2000; Upstate Biotechnology, USA) diluted in Trizma base solution containing 0.3% Triton X-100 (TTBS) with 50 mM NaF and 3% BSA, overnight at room temperature with gentle shaking. Again sections were kept covered. Sections were then washed several times in PB and incubated in biotinylated goat anti-rabbit secondary antibody (diluted 1:200 in TTBS; Vectastain, Vector Laboratories, Burlingame, CA, USA) and 1.5% normal goat serum for 2 h at room temperature on a rotator. Sections were then washed in PB and processed with avidin-biotinylated horseradish peroxidase complex in TTBS (Elite Kit, Vector Laboratories) for 1 h at room temperature, again with constant rotation, and then covered. Sections were washed again in PB and then the reaction was visualized using diaminobenzidine (DAB Substrate Kit, Vector Laboratories). The reaction was stopped by washing in cold PBS, and then the sections were mounted on gelatine-coated slides and cover-slipped.

*Cell counts and statistics:* Wherever possible, the experimenter was blind to the group identity of each animal. In addition, all counts used a standardized procedure so that the threshold was set automatically, based on the overall intensity of the image. For each brain area, counts of immunopositive cells were taken from at least three sections from each hemisphere and the six or more counts then averaged to produce a mean score for each animal. The analyses matched those for Experiment 1 except there were no additional factors relating to behavioral protocols. All rats were paired immunohistochemically and analyzed as pairs using their cell counts data (as in Experiment 1). Pair-data were not, however, then normalized (unlike Experiment 1) as all rats received the same behavioral experiences, i.e., there was not the additional factor of training condition. Counts from the retrosplenial cortex were again subdivided between superficial (I to upper III) and deep (mid III–VI) layers, in response to previous findings that changes in c-*fos* activity after anterior thalamic lesions are most pronounced in the superficial layers of the granular retrosplenial cortex (Jenkins et al., 2004b; Poirier et al., 2008a,b; Poirier and Aggleton, 2009). In addition, separate analyses were conducted for just the granular retrosplenial cortex following the finding from Experiment 1 that *zif268* changes were most apparent in this subdivision of the cortical area.

### Results

#### Bilateral anterior thalamic lesions

**Fig. 5:**
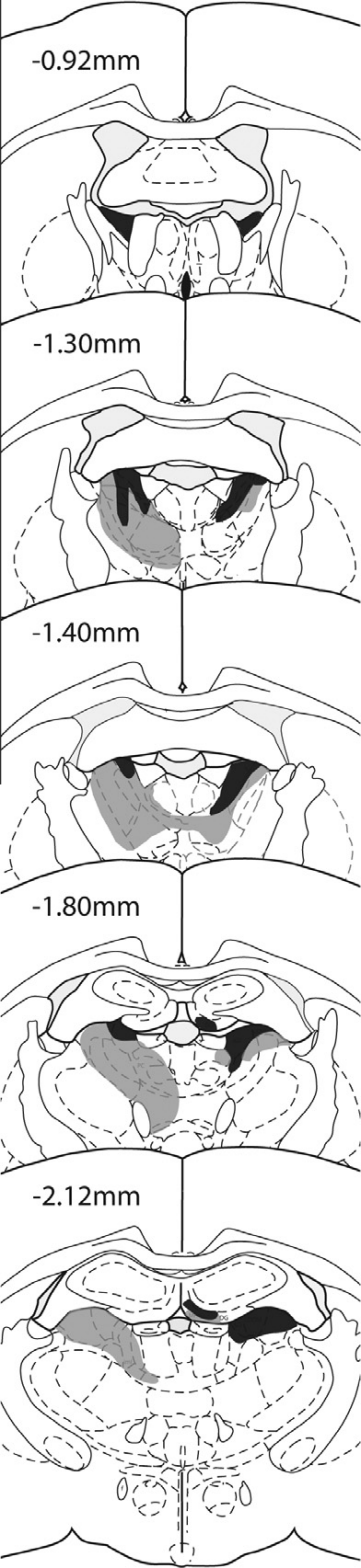
Experiment 2. Coronal sections depicting the cases with the smallest (dark gray) and largest (light gray) bilateral anterior thalamic lesions. The numbers refer to the approximate distance of the section in mm caudal to bregma. The sections are modified from Paxinos and Watson (2005).

Of the seven rats with anterior thalamic lesions, one was excluded as the thalamic lesions were unusually small. The anterior thalamic lesions in the remaining six rats consistently produced appreciable degrees of cell loss in the anterior dorsal and anterior ventral thalamic nuclei (Fig. 5). In every case the anterior thalamic nuclei were clearly shrunken in all three planes as a result of the cell loss. The respective mean tissue loss across the anterior thalamic nuclei in these rats was 39.2%, with a median score of 42% (range 23.2–61.7%). The anterior dorsal nucleus was the most consistently atrophied, while the anterior medial nucleus showed the greatest degree of sparing (Fig. 5). Other rostral thalamic nuclei were left intact, although unilateral damage to the dorsal part of the rostral portion of the lateral dorsal nucleus was seen in five cases, with bilateral damage in the same region in one further case. Some very restricted cell loss was sometimes present at the rostral limit of the very medial margin of the septal hippocampus. This very restricted cell loss, which included parts of the medial blade of the dentate gyrus, was unilateral in three cases and bilateral in three cases. The final group sizes were; ATN2 *n* = 6, Sham2 *n* = 6.

#### Radial-arm maze behavior

Although all animals were forced to visit the same arms in the same order, there were individual differences in the total numbers of arms visited within the 20-min time period. The two groups did not differ on the total numbers of arms visited [mean ATN2 = 16.0 (sd 6.9), Sham2 = 14.2 (sd 4.3), *p* > 0.1].

#### Immunohistochemical (*zif268*, CREB, PCREB, GAP-43) results

**Fig. 6:**
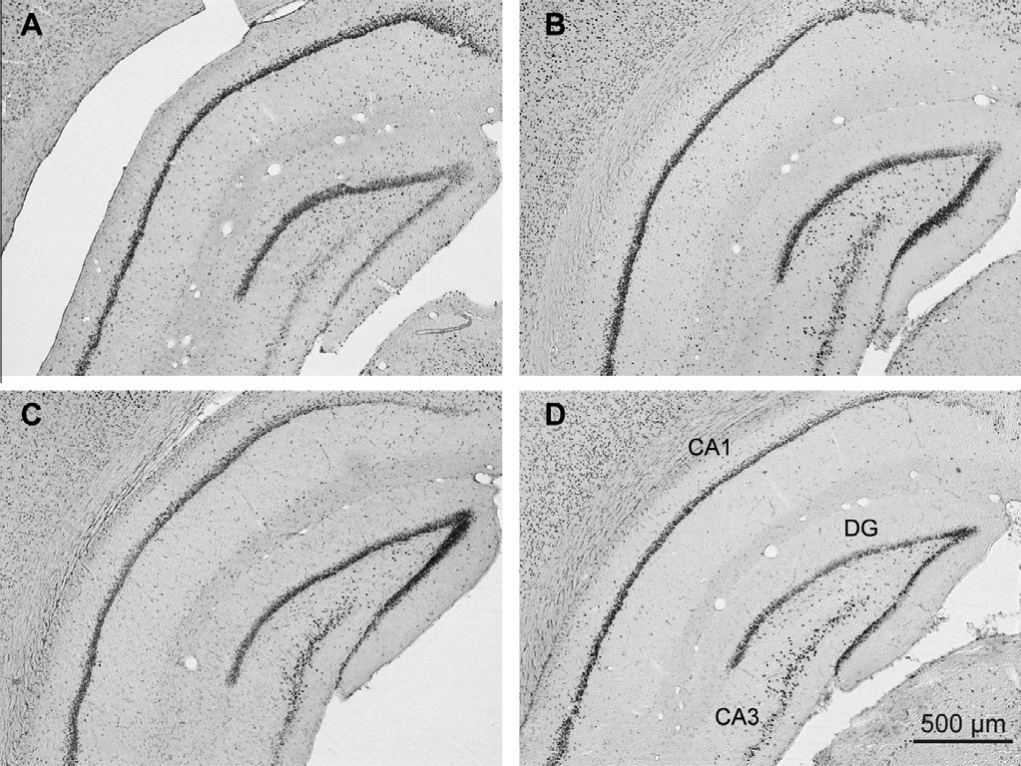
Photomicrographs (bright field) of coronal sections from the dorsal (intermediate) hippocampus showing immunohistochemically stained cells for CREB (upper; A, B) and pCREB (lower; C, D). Sections from the Control group are in the left column (A, C), while sections from the group with anterior thalamic lesions (ATN) are in the right column (B, D). *Abbreviations used in the figures:* DG, dentate gyrus.

For the various regions within each grouping there were often significant differences in the raw cell counts for a given marker. These changes are only reported if they appear to be affected by the thalamic lesion. Because of the close relationship between CREB and pCREB (Fig. 6), we also compared their scores in combined analyses for those sites where there was evidence of a lesion effect for one or both of the molecules.

##### Hippocampal subfields

**Fig. 7:**
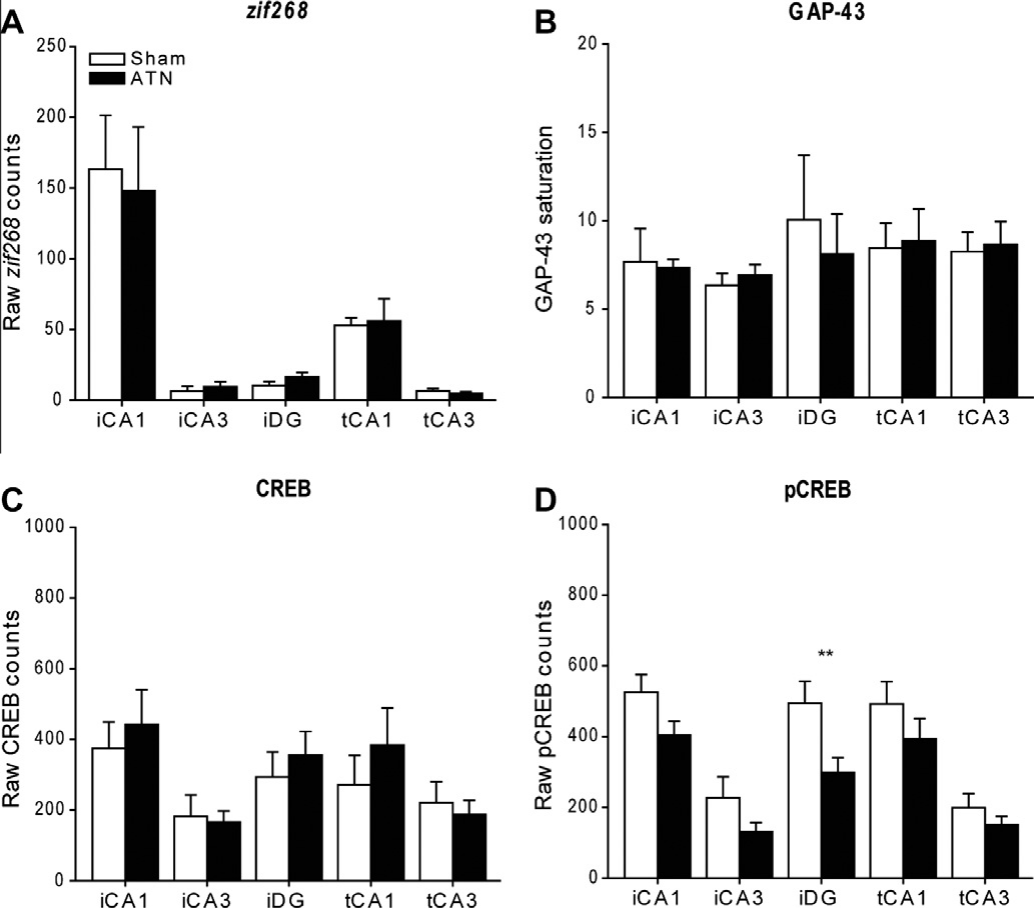
Hippocampus: Counts of immunopositive cells in the five hippocampal subregions in the Sham and anterior thalamic lesion (ATN) groups. Data (mean ± standard error of the mean) are shown for: (A) *zif268*, (B) GAP-43, (C) CREB, and (D) pCREB. *Abbreviations used in the figures:* iCA1, intermediate CA1; iCA3, intermediate CA3; iDG, intermediate dentate gyrus; tCA, temporal CA1; tCA3, temporal CA3. ^∗∗^*p* < 0.01.

*zif268:* The first analyses compared the separate cell counts taken in the five subfields of the hippocampus; dorsal (intermediate) CA1, dorsal (intermediate) CA3, dorsal (intermediate) dentate gyrus, ventral (temporal) CA1 and ventral (temporal) CA3. There was no group difference (*p* > 0.1) and no group by subfield interaction (*p* > 0.1; Fig. 7A).

*GAP-43:* There was no evidence of a difference in GAP-43 counts between the two group (*p* > 0.1), neither was there a group by region interaction (*p* > 0.1; Fig. 7B).

*CREB:* There was no evidence that the rats with anterior thalamic lesions showed a consistent change in CREB levels across the hippocampus (*p* > 0.1) and there was no group by area interaction (*p* > 0.1; Figs. 6, 7C).

*pCREB:* Overall, there appeared to be a reduction in pCREB cell-positive counts in the rats with anterior thalamic lesions (Figs. 6, 7D), and this change was significant (*F*_(1, 10)_ = 9.04, *p* = 0.013). No group by area interaction was observed (*p* > 0.1). The simple effects indicated that pCREB levels were reduced in the intermediate dentate gyrus (*F*_(1, 50)_ = 8.18, *p* = 0.006).

*pCREB versus CREB:* An analysis of variance (two within and one between factor) helped to confirm the different impact of anterior thalamic damage upon these two molecules. There was a significant interaction between the levels of these two proteins in the hippocampus as only pCREB decreased after anterior thalamic lesions (*F*_(1, 10)_ = 7.09, *p* = 0.024). The three-way interaction (*F*_(4, 40)_ = 2.66, *p* = 0.047) reflected the finding that this differential change was larger for some regions (CA1, dentate gyrus) than others (CA3).

##### Subicular and entorhinal cortices

**Fig. 8:**
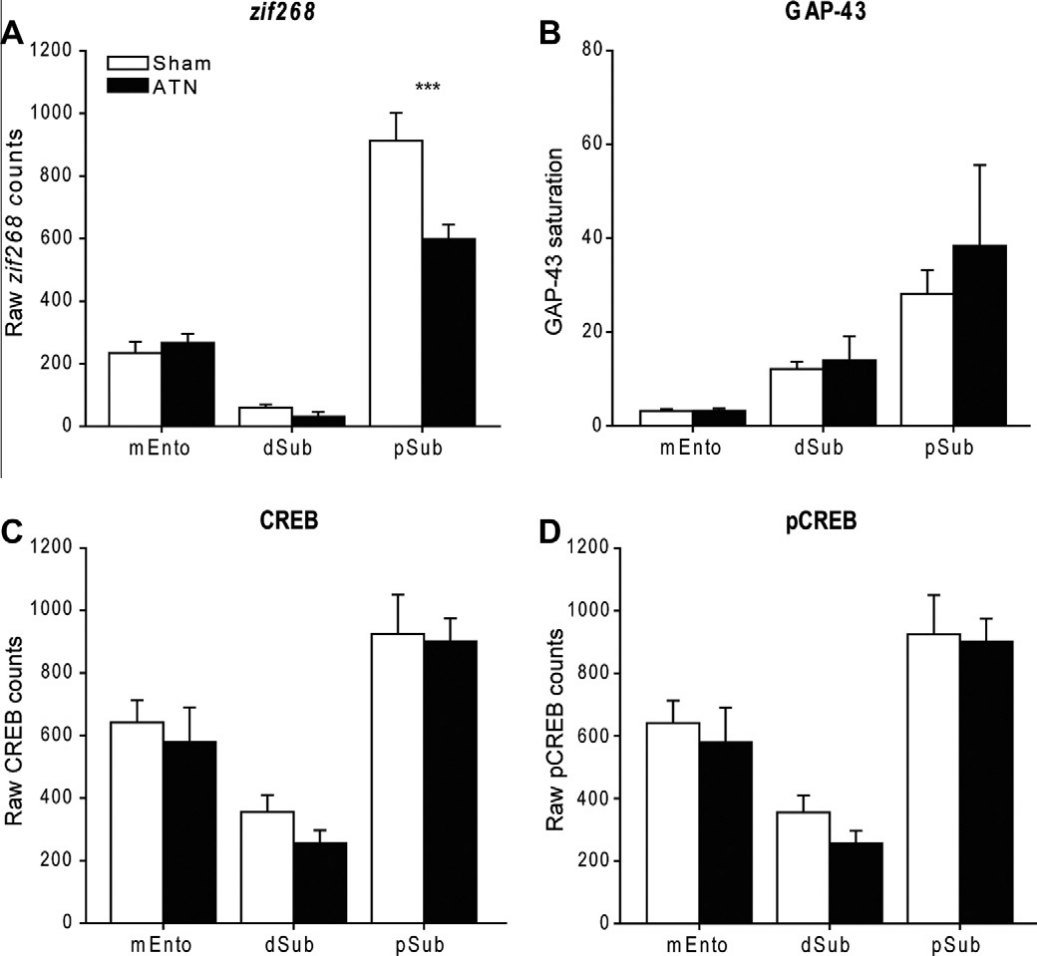
Subicular and entorhinal cortices: Counts of immunopositive cells in the subicular and entorhinal cortices in the Sham and anterior thalamic lesion (ATN) groups. Data (mean ± standard error of the mean) are shown for: (A) *zif268*, (B) GAP-43, (C) CREB, and (D) pCREB. *Abbreviations used in the figures:* dSub, dorsal subiculum; mEnto, medial entorhinal cortex; pSub, postsubiculum. ^∗∗∗^*p* < 0.001.

*zif268:* The first analyses compared the separate cell counts taken in the postsubiculum, dorsal subiculum, and medial entorhinal cortex. Overall the ATN group had lower *zif268* cell-positive counts (*F*_(1, 10)_ = 7.18, *p* = 0.023), but this effect was region specific as shown by the group by subfield interaction (*F*_(2, 20)_ = 8.56, *p* = 0.002). Simple effects showed that the significant impact of the thalamic lesions was confined to the postsubiculum, where the lesions produced a large drop in *zif268* positive cells (*F*_(1, 30)_ = 23.73, *p* < 0.001; Fig. 8A).

*GAP-43:* There was no evidence of a thalamic lesion effect (*p* > 0.1) and no lesion by region interaction (*p* > 0.1; Fig. 8B).

*CREB:* Likewise, there was no evidence of a lesion group difference in the numbers of CREB-positive cells (*p* > 0.1) nor any group by area interaction (*p* > 0.1; Fig. 8C).

*pCREB:* Again, no evidence was found that the lesions affected overall cell-positive counts across the three areas (*p* > 0.1), and no group by area interaction was observed (*p* > 0.1; Fig. 8D).

##### Retrosplenial cortex

Counts were first made in the same five subfields as in Experiment 1. Additional analyses focused on the granular retrosplenial cortex as this subdivision showed consistent *zif268* changes in Experiment 1 following anterior thalamic damage.

**Fig. 9:**
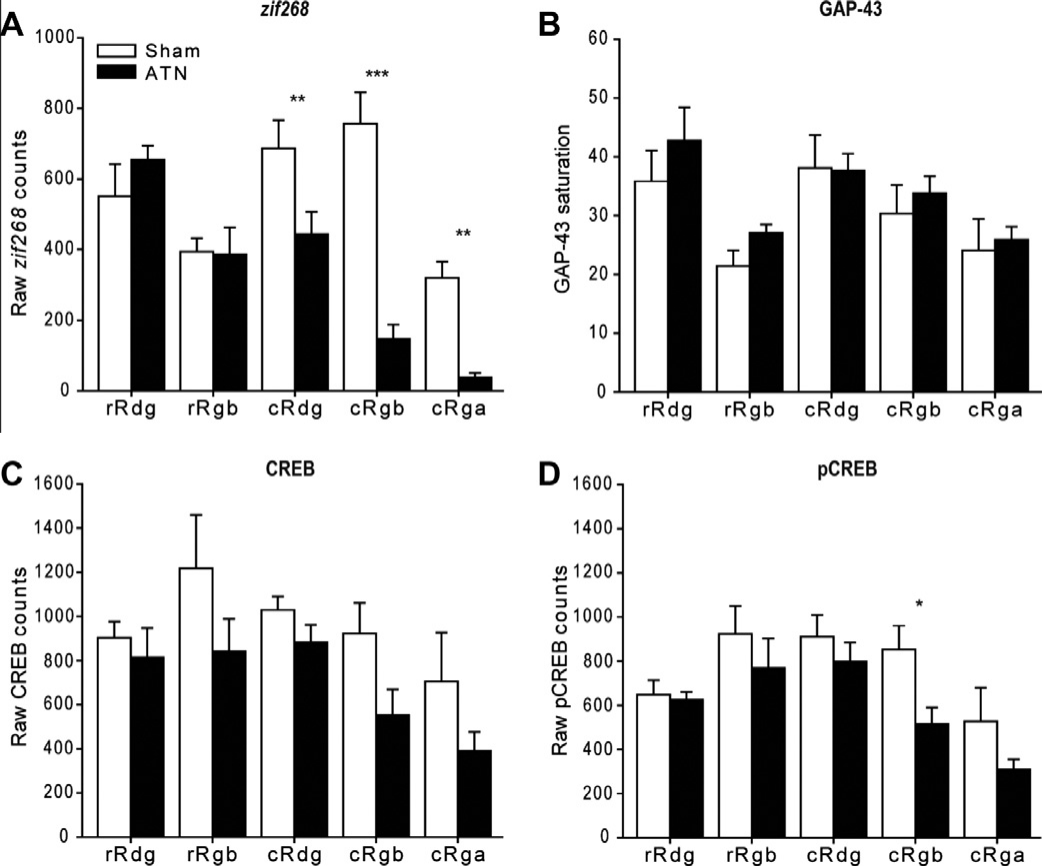
Retrosplenial cortices: Counts of immunopositive cells in the five retrosplenial subregions in the Sham and anterior thalamic lesion (ATN) groups. Data (mean ± standard error of the mean) are shown for: (A) *zif268*, (B) GAP-43, (C) CREB, and (D) pCREB. *Abbreviations:* cRdg, caudal dysgranular retrosplenial cortex; cRga, caudal granular retrosplenial cortex, area a; cRgb, caudal granular retrosplenial cortex, area b; rRdg, rostral dysgranular retrosplenial cortex; rRgb, rostral granular retrosplenial cortex, area b. ^∗^*p* < 0.05; ^∗∗^*p* < 0.01; ^∗∗∗^*p* < 0.001.

*zif268:* Overall, the ATN group had lower *zif268* cell-positive counts (*F*_(1, 10)_ = 8.35, *p* = 0.016) but this effect was selective as shown by the group by subfield interaction (*F*_(4, 40)_ = 22.4, *p* < 0.001; Fig. 9A). Simple effects showed that the thalamic lesions produced a significant drop in *zif268* positive cells in caudal granular retrosplenial cortex, area a (cRga), caudal granular retrosplenial cortex, area b (cRgb), cRdg (all *p* < 0.01), i.e., in caudal parts of the retrosplenial cortex. Analyses of the three granular subfields revealed a significant interaction between layer (superficial or deep) and surgical condition (*F*_(1, 10)_ = 8.63, *p* = 0.015) reflecting the greater impact of the thalamic surgeries on the more superficial granular retrosplenial cortex.

*GAP-43:* There was no evidence of a group difference in the numbers of GAP-43 positive cells (*p* > 0.1) or any group by subfield interaction (*p* > 0.1; Fig. 9B). Likewise, there was no evidence of a difference between the sensitivity of superficial and deep cortical layers (*p* > 0.1).

*CREB:* There were no significant lesion related changes in CREB positive cells across the five subfields that comprised the area (*F*_(1, 10)_ = 3.16, *p* = 0.106) and no group by subfield interaction (*p* > 0.1; Fig. 9C). Additional CREB analyses focused on just the granular (rostral Rgb, caudal Rgb, Rga) retrosplenial cortex for both superficial and deep layers. While mean CREB levels appeared reduced following anterior thalamic lesions, this change was not significant (*p* > 0.1) and there was no interaction with region or layers (superficial or deep; *p* > 0.1 for both).

*pCREB:* Although there were signs of a reduction in pCREB-positive cells across the region, this effect was not significant (*F*_(1, 10)_ = 4.24, *p* = 0.067) and there was no group by area interaction (*p* > 0.1; Fig. 9D). Inspection of the simple effects indicated that ATN lesions significantly reduced the pCREB counts in caudal Rgb (*F*_(1, 50)_ = 5.70, *p* = 0.021).

Again, in view of the consistent *zif268* changes in granular retrosplenial cortex in both Experiments 1 and 2, additional pCREB analyses examined just the granular (rostral Rgb, caudal Rgb, Rga) cortex for both superficial and deep retrosplenial layers. A significant reduction in pCREB was found following anterior thalamic lesions (*F*_(1, 10)_ = 7.15 *p* = 0.023), though there was no interaction with region (*p* > 0.1) as the reduction was not disproportionate in any single area. There were also significantly more pCREB counts in the deep compared with superficial layers (*F*_(1, 10)_ = 9.64, *p* = 0.011), but there was no interaction with either region or group (*p* > 0.1). Simple effects revealed a significant fall in pCREB in the superficial part of caudal Rgb (*F*_(1, 60)_ = 4.37, *p* = 0.041). When the pCREB and CREB data were analyzed together there was no evidence of an interaction (*p* > 0.1) as levels of the two markers did not diverge.

##### Prefrontal cortex

**Fig. 10:**
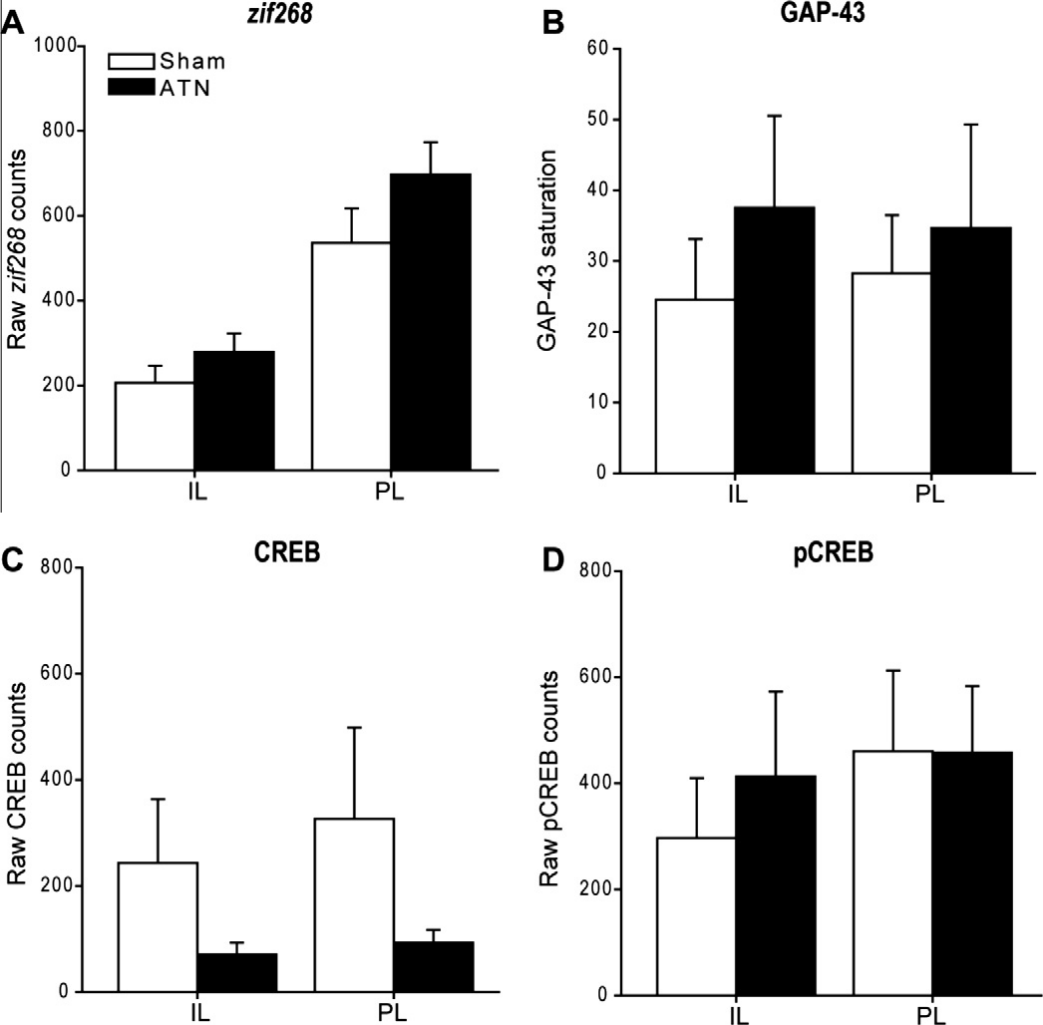
Prefrontal cortex: Counts of immunopositive cells in the infralimbic cortex (IL) and prelimbic cortex (PL) in the Sham and anterior thalamic lesion (ATN) groups. Data (mean ± standard error of the mean) are shown for: (A) *zif268*, (B) GAP-43, (C) CREB, and (D) pCREB.

*zif268:* Overall, the ATN group appeared to have higher *zif268*-positive cell counts although this change was not significant (*p* > 0.1; Fig. 10A).

*GAP-43:* While there was no overall change in GAP-43-positive cells between the two groups (*p* > 0.1), there was a group by area interaction (*F*_(1, 10)_ = 7.81; *p* = 0.019). This interaction reflected the slight increase in counts for the Sham group going from IL to PL (*F*_(1, 10)_ = 5.05, *p* = 0.049), a pattern not seen in the ATN2 group (Fig. 10B).

*CREB:* Although the mean counts of CREB-positive cells appeared lower in the ATN group, this change was not significant (*p* > 0.1) and there was no interaction (*p* > 0.1; Fig. 10C).

*pCREB:* There was no evidence of a change in the numbers of pCREB-positive cells across the two areas (*p* > 0.1; Fig. 10D).

##### Control cortex (primary auditory cortex)

Analyses based on the raw cell counts found no evidence of a difference between the Sham2 and ATN2 groups for either *zif268*, GAP-43, CREB, or pCREB (all *p* ⩾ 0.1).

## Discussion

Lesions were placed in the anterior thalamic nuclei of rats in two experiments. Measurements were then made of several molecules linked to neuronal plasticity in a set of limbic sites related to anterior thalamic function. In Experiment 1, rats were exposed to either novel or familiar objects prior to analysis of the immediate-early gene *zif268*, and so the experiment included sites presumed to be involved in object recognition memory. In Experiment 2, all rats were exposed to novel room cues in a radial-arm maze, and so the study included sites thought to be involved in spatial learning. Experiment 2 examined CREB, pCREB, and GAP-43, in addition to *zif268*. Given the various differences between Experiments 1 and 2, any changes in *zif268* that were common to both experiments are presumably highly reliable. In fact, the most reliable changes were in the granular retrosplenial cortex (area 29) where anterior thalamic lesions consistently reduced *zif268* activity, irrespective of whether the lesion was unilateral or bilateral, the strain of rat, or the behavioral task that the rat had performed immediately prior to IEG measurement (see also Jenkins et al., 2004b; Poirier and Aggleton, 2009). In addition to the retrosplenial cortex, the postsubiculum also showed reduced *zif268* activity following anterior thalamic lesions in both experiments. No consistent *zif268* changes were found in any of the other sites studied, despite the fact that most of the sites examined have direct connections with the anterior thalamic nuclei (Shibata 1992, 1993a,b).

A central feature of the present study was the analysis of rats with unilateral thalamic lesions in Experiment 1 and bilateral anterior thalamic lesions in Experiment 2. This design was selected because of inherent shortcomings with either approach. Unilateral lesions (Experiment 1) have the advantage that all comparisons can be made across hemispheres within the same animal, i.e., they are exceptionally well controlled for perceptual-motor factors, but there is the risk that null results might reflect fibers that cross between the hemispheres. Bilateral anterior thalamic lesions help minimize the impact of any crossing fibers but, unlike unilateral lesions, they are sufficient to induce marked learning changes, e.g., to spatial learning (Warburton et al., 2001; Aggleton et al., 2009). As a consequence, there might be chronic changes in the animals’ behavior that could lead to alterations in the status of limbic structures. Consequently, there remains the possibility of changes to structures, such as the hippocampus, that are secondary, rather than primary. It is this possibility that should be excluded by investigating rats with unilateral thalamic lesions.

An integral part of Experiment 1 was the contrast between rats that had just explored novel objects and rats that had just explored familiar objects. Advantages associated with the bow-tie maze protocol include the ability to give each rat multiple trials within a session, yet without the need to handle the rat between trials (Albasser et al., 2010a). One potential disadvantage was that the overall times spent exploring objects could not be fully matched on the final session between Group Novel and Group Familiar. This time difference arose as an almost inevitable consequence of repeating the same objects across all previous sessions for Group Familiar, i.e., the resultant decreases in spontaneous exploration confirmed that the rats in this group correctly perceived the repeated objects as familiar. In order to match total exploration times across the two treatment groups it would have been necessary to give Group Familiar extra trials with additional objects, but this would introduce other differences. With these considerations in mind, it can be noted that the baiting procedure should help to maintain comparable patterns of behavior across the two groups, i.e., rats approached and manipulated all objects.

A previous study using the same behavioral design as Experiment 1 found that novel stimuli raised c-*fos* activity in caudal perirhinal cortex, area TE2, and hippocampal subfields CA3 and CA1, while the dentate gyrus decreased its c-*fos* activity (Albasser et al., 2010b). The present study extended this protocol to measure *zif268* responses. While some differential hippocampal *zif268* activity was associated with the novel versus familiar stimuli, this effect appeared localized. In particular, temporal CA1 showed a relative increase in *zif268* activity associated with novel objects compared with temporal CA3, which showed a decrease. These changes contrasted with a lack of any differential *zif268* changes in the perirhinal cortex, despite the known importance of this cortical area for object recognition whether tested in an open arena or in a bow-tie maze (Ennaceur et al., 1996; Winters et al., 2008; Albasser et al., 2010a). In fact, the current null result for *zif268* in the perirhinal cortex is not unexpected as earlier studies had found c-*fos*, but not *zif268*, activity changes in the perirhinal cortex when rats are shown novel visual stimuli (Brown and Xiang, 1998; Wan et al., 1999; Aggleton et al., 2012; see also Romero-Grandados et al., 2010). The implication is that *zif268* activity in the perirhinal cortex is not a key process for effective long-term object recognition memory (although see Jones et al., 2001; Bozon et al., 2003). In contrast, the functional significance of the perirhinal c-*fos* response for object recognition memory has recently been demonstrated by blocking c-*fos* activity in this cortical area, and finding that this manipulation impairs recognition memory after extended retention delays (Seoane et al., 2012). There remains a concern that the relatively localized changes in *zif268* activity associated with the behavioral tasks in Experiment 1 may have reduced the sensitivity of the protocol for looking at lesion effects. While this concern cannot be dismissed, it should be noted that the raw *zif268* counts in all hippocampal areas examined remained high and that local differential hippocampal responses (e.g., in temporal CA1) were identified. Consequently, there remained the potential for seeing lesion-induced changes in hippocampal *zif268*, but they were not observed.

As noted, some hippocampal subfields show differential c-*fos* (Albasser et al., 2010b) and *zif268* (present study) responses to novel versus familiar stimuli when tested in the bow-tie maze. These findings might suggest that the rodent hippocampus has a direct role in supporting object recognition memory. This conclusion should, however, be treated with caution. The first reason is that extensive hippocampal lesions do not appear to alter object recognition memory when it is tested in the bow-tie maze, even when using a range of retention intervals (Albasser et al., 2010a, 2012). A second reason is that on encountering a novel object, rats will spontaneously learn much about its associative features, e.g., its spatial and temporal location. This spontaneous associative learning is hippocampal dependent (Save et al., 1992; Ennaceur et al., 1997; Mumby et al., 2002; Piterkin et al., 2008; Warburton and Brown, 2010; Barker and Warburton, 2011; Albasser et al., 2012) and is consistently associated with changes in hippocampal c-*fos* activity (Wan et al., 1999; Jenkins et al., 2004a; Amin et al., 2006). Given these results, the local changes in hippocampal *zif268* found in this behavioral task are not unexpected and need not directly reflect object recognition.

The major finding from Experiment 2 concerned the relative changes in CREB and pCREB levels in the hippocampus following anterior thalamic damage. While hippocampal pCREB levels were significantly lower in those rats with anterior thalamic lesions compared to the Sham2 rats, hippocampal CREB levels did not show this pattern. Consequently there was a significant interaction as CREB and pCREB showed different relative profiles. These findings are notable because the conversion of CREB to pCREB within the hippocampus is seen as a key step for consolidating spatial learning (Guzowski and McGaugh, 1997; Guzowski, 2002; Mizuno et al., 2002; Winograd and Viola, 2004). Consequently, the profile of results in the ATN2 rats suggests a failure of this conversion mechanism within the hippocampus, at least when rats are placed in the radial-arm maze. This finding is potentially revealing as it is already known that CREB phosphorylation in the hippocampus correlates with learning in the radial-arm maze (Mizuno et al., 2002), and that both hippocampal lesions and anterior thalamic lesions impair spatial learning in this same apparatus (Olton et al., 1979; Aggleton et al., 1996; Warburton et al., 2001). Furthermore, cross-lesion disconnections have shown that the hippocampus and anterior thalamic nuclei have interdependent roles in supporting spatial learning, including radial-arm maze learning (Warburton et al., 2001; see also Olton et al., 1982; Henry et al., 2004). It would, therefore, appear that CREB phosphorylation in the hippocampus is under the partial control of the anterior thalamic nuclei. Such a mechanism might be expected to affect principally the long-term maintenance of spatial information (Guzowski and McGaugh, 1997).

To appreciate the wider implications of this finding is it useful to note that the conversion of CREB to pCREB is a key step in learning-induced plasticity that includes the regulation of various IEGs, including c-*fos* and *zif268* (Guzowski and McGaugh, 1997; Silva et al., 1998; Guzowski, 2002; Winograd and Viola, 2004; Countryman et al., 2005). Consistent with this mechanism, previous studies have found that anterior thalamic lesions reliably reduce hippocampal c-*fos* activity (Jenkins et al., 2002a,b). This c-*fos* hypoactivity could, therefore, reflect changes in CREB phosphorylation associated with anterior thalamic damage, although it must be selective in that *zif268* levels are not equally affected. Further support for the notion that hippocampal IEG disruption (e.g., of c-*fos*) contributes to the functional deficits after anterior thalamic lesions comes from the finding that normal rats placed in a radial-arm maze surrounded by novel spatial cues (as in the present study) show increased c-*fos* activity both in the hippocampus and anterior thalamus (Vann et al., 2000; Jenkins et al., 2002b). In addition, blockade of hippocampal c-*fos* activity disrupts radial-arm maze performance (He et al., 2002) and the re-activation of hippocampal c-*fos* positive cells can seemingly resurrect spatial memories (Liu et al., 2012). While the same task as that used in the present study (radial-arm maze in a novel room) does not appear to have been used before to measure *zif268* activity, it has been found that performance of a spatial working memory task in the radial-arm maze can correlate with hippocampal *zif268* activity (Poirier et al., 2008a). Other studies also indicate a role for *zif268* in maintaining spatial information (e.g., Bozon et al., 2002). Thus, it can be seen that the current evidence suggests that the changes in hippocampal CREB phosphorylation following anterior thalamic damage disrupt several pathways, including c-*fos* expression, that then impair spatial learning. While this description does not preclude the additional involvement of other IEGs that are also regulated by CREB, including *zif268* (Guzowski, 2002), the lack of any thalamic lesion-induced changes in hippocampal *zif268* indicates that this IEG is principally under the control of other sites.

This description, that anterior thalamic damage impairs spatial learning via its impact on hippocampal function, including CREB/pCREB regulation, offers only one level of analysis. This account does not explain functionally why, how, or when the anterior thalamic nuclei might regulate hippocampal activity. A second issue is that the anterior thalamic lesions in Experiment 2 sometimes produced a very restricted zone of cell loss at the medial margin of the most rostral part of the septal hippocampus that could potentially affect local hippocampal activity. Additional inter-hemispheric comparisons for hippocampal CREB and pCREB were, therefore, conducted between the hemisphere that was either intact (or had minimal cell loss) versus the hemisphere with the greater cell loss (albeit very restricted). No differences were apparent for hippocampal CREB or pCREB (both *p* > 0.1). It should also be added that all hippocampal measurements were taken far away from any sites of possible pathology. An opposite concern is that the anterior thalamic damage in Experiment 2 was typically more restricted than that in Experiment 1 and the group sizes modest, so the findings may underestimate the extent of distal dysfunction. While this may be true, the same thalamic lesions were sufficient to severely impair T-maze alternation (Aggleton et al., 2009). A third issue is that only one time period post surgery was examined, and so it cannot be determined if the CREB/pCREB imbalance takes time to develop. It is, however, known that anterior thalamic lesions impair spatial memory from the very first post-operative session (Warburton et al., 1999) and that c-*fos* hypoactivity is present in the retrosplenial cortex from the earliest time point so far measured, 1 week after surgery, to at least 1 year after surgery (Poirier and Aggleton, 2009).

The impact of the anterior thalamic lesions upon hippocampal CREB/pCREB balance could be via the loss of direct connections between the two structures or via indirect connections, such as those through the retrosplenial cortex. The latter proposal receives some support from the marked IEG changes seen in the retrosplenial cortex for c-*fos* (Jenkins et al., 2002a, 2004b; Poirier et al., 2008b; Poirier and Aggleton, 2009) and *zif268* (Jenkins et al., 2004b; also present study). These combined IEG changes strongly predict that the retrosplenial cortex should also show alterations in CREB/pCREB activity. While counts involving all retrosplenial regions failed to support this prediction, a significant reduction of pCREB was found when the analyses targeted just the granular retrosplenial regions, i.e., those regions showing the most consistent IEG changes following anterior thalamic damage (Jenkins et al., 2002a, 2004b; Poirier and Aggleton, 2009). The same retrosplenial subregions show a loss of neural plasticity, as demonstrated by electrophysiological stimulation of retrosplenial tissue after anterior thalamic lesions (Garden et al., 2009). Unlike the hippocampus, no interaction between retrosplenial CREB and pCREB activity was found in the present study as levels of both markers tended to decline, although this was not significant for CREB. This overall pattern of CREB and pCREB results suggests that there could be indirect effects (via the retrosplenial cortex) as well as direct effects, upon the hippocampus following anterior thalamic damage.

Experiment 2 also looked at levels of the GAP-43 protein. GAP-43 is thought to regulate axonal growth during development, including the targeting of other neurons for synapse formation (Benowitz and Routtenberg, 1997; Wehrle et al., 2001). The same protein is transiently upregulated after axotomy of CNS neurons and is, thereby, thought to be involved in axonal regeneration after damage in the adult brain. A consequence is that the sprouting of axotomized neurons is often associated with GAP-43 expression in the developing and adult brain (Benowitz and Routtenberg, 1997; Wehrle et al., 2001). Other evidence suggests that GAP-43 is involved in the signaling pathway that provokes the decision for either axonal growth or cell death (retraction), i.e., opposite cellular outcomes (Wehrle et al., 2001). For these reasons, particular interest focused on the retrosplenial cortex, not only because this area receives dense direct inputs from the anterior thalamic nuclei (van Groen and Wyss, 1990, 1992, 2003) but also because anterior thalamic lesions reliably disrupt the expression of IEGs such as c-*fos* and *zif268* within the retrosplenial cortex (Jenkins et al., 2004b; Poirier and Aggleton, 2009). Indeed, gene array studies show that the retrosplenial changes after anterior thalamic lesions are pervasive, affecting a wide variety of other genes encoding transcription factors, including *brd8*, *fra-2*, *klf5*, *nfix*, *nr4a1*, *smad3*, *smarcc2* and *zfp9* (Poirier et al., 2008b). In contrast, cell numbers seem little affected in the retrosplenial cortex after anterior thalamic damage (Jenkins et al., 2004b; Poirier and Aggleton, 2009), raising the question of whether there might be more subtle white matter changes. A complex array of changes could, therefore, have occurred in the retrosplenial cortex in response to the thalamic surgeries, e.g., sprouting from within the cortex (Carmichael et al., 2005) as well as the retraction of afferents. In fact, the present study found no convincing evidence of changes in GAP-43 within the retrosplenial cortex although this null result can only be regarded as preliminary until a range of post-surgical intervals are examined (see also Steward, 1995).

In summary, the present study helps to highlight the special three-way relationship between the anterior thalamic nuclei, the retrosplenial cortex, and the hippocampus (Albasser et al., 2007; Vann et al., 2009). Not only are all three areas directly interconnected but they are also thought to function together to support spatial learning and memory (Sutherland and Rodriguez, 1989; Vann et al., 2009). This inter-relationship appears to not only include the expression of IEGs such as c-*fos* and *zif268*, but also how they are regulated by pCREB. Consequently, the present findings add additional support to the concept of an ‘extended hippocampal system’ (Aggleton and Brown, 1999) in which hippocampal function is dependent on distal sites such as the anterior thalamus and retrosplenial cortex.

## Figures and Tables

**Table 1:** Presentation order of objects for Group Familiar (top) and Group Novel (bottom) for Session 1 (left) and Session 13 (final; right). Each of the 13 sessions contains 20 trials. Each trial has two objects depicted by a letter with the exception of Trial 0, which allows the first object to become familiar. For Group Novel, different sets of objects were used throughout training, and novel objects are indicated in bold type. For Group Familiar, the same set of objects was used for every session, and so the less recently experienced items are indicated in bold type. The identity of the objects was the same for Group Familiar and Group Novel on only the final session

	Session 1								Session 13 (final)						
*Group Familiar*															
Trials	0	1	2	3	4	5	6–20		0	1	2	3	4	5	6–20
Objects	–	A	B	C	D	E	…	Sessions 2–12	–	C	F	B	E	D	…
	**A**	**B**	**C**	**D**	**E**	**F**	**…**		**C**	**F**	**B**	**E**	**D**	**A**	**…**

*Group Novel*															
Trials	0	1	2	3	4	5	6–20		0	1	2	3	4	5	6–20
Objects	–	Ψ	Ω	α	β	γ	…	Sessions 2–12	–	C	F	B	E	D	…
	**Ψ**	**Ω**	**α**	**β**	**γ**	**ε**	**…**		**C**	**F**	**B**	**E**	**D**	**A**	**…**
